# Cultivar-Based Introgression Mapping Reveals Wild Species-Derived *Pm-0*, the Major Powdery Mildew Resistance Locus in Squash

**DOI:** 10.1371/journal.pone.0167715

**Published:** 2016-12-09

**Authors:** William L. Holdsworth, Kyle E. LaPlant, Duane C. Bell, Molly M. Jahn, Michael Mazourek

**Affiliations:** 1 Section of Plant Breeding and Genetics, School of Integrative Plant Sciences, Cornell University, Ithaca, NY, United States of America; 2 Rupp Seeds, Inc., Wauseon, OH, United States of America; 3 Department of Agronomy, University of Wisconsin-Madison, Madison, WI, United States of America; US Department of Agriculture, UNITED STATES

## Abstract

Powdery mildew is a major fungal disease on squash and pumpkin (*Cucurbita* spp.) in the US and throughout the world. Genetic resistance to the disease is not known to occur naturally within *Cucurbita pepo* and only infrequently in *Cucurbita moschata*, but has been achieved in both species through the introgression of a major resistance gene from the wild species *Cucurbita okeechobeensis* subsp. *martinezii*. At present, this gene, *Pm-0*, is used extensively in breeding, and is found in nearly all powdery mildew-resistant *C*. *pepo* and *C*. *moschata* commercial cultivars. In this study, we mapped *C*. *okeechobeensis* subsp. *martinezii*-derived single nucleotide polymorphism (SNP) alleles in a set of taxonomically and morphologically diverse and resistant *C*. *pepo* and *C*. *moschata* cultivars bred at Cornell University that, by common possession of *Pm-0*, form a shared-trait introgression panel. High marker density was achieved using genotyping-by-sequencing, which yielded over 50,000 *de novo* SNP markers in each of the three *Cucurbita* species genotyped. A single 516.4 kb wild-derived introgression was present in all of the resistant cultivars and absent in a diverse set of heirlooms that predated the *Pm-0* introgression. The contribution of this interval to powdery mildew resistance was confirmed by association mapping in a *C*. *pepo* cultivar panel that included the Cornell lines, heirlooms, and 68 additional *C*. *pepo* cultivars and with an independent F_2_ population derived from *C*. *okeechobeensis* subsp. *martinezii* x *C*. *moschata*. The interval was refined to a final candidate interval of 76.4 kb and CAPS markers were developed inside this interval to facilitate marker-assisted selection.

## Introduction

Powdery mildew, caused by the obligate biotrophic pathogens *Podosphaera xanthii* and *Golovinomyces cichoracearum*, is one of the most prevalent and destructive fungal diseases globally of *Cucurbita* species, and especially of *C*. *pepo*, the most economically important species of squash and pumpkin [1–5]. In the US, *P*. *xanthii* (syn. *Podosphaera fusca*, *Sphaerotheca fuliginea*) is the most common powdery mildew pathogen species on *Cucurbita* [6]. *P*. *xanthii* can infect numerous species in the Asteraceae, Cucurbitaceae, Lamiaceae, Scrophulariaceae, Solanaceae, and Verbenaceae families and is easily spread between hosts via windborne asexual conidia [7, 8]. Powdery mildew on squash and pumpkin is easily identified by white mycelial growth on stems, petioles, and leaf surfaces that appear four to seven days post-infection [9]. Symptoms include chlorotic lesions that can eventually lead to whole plant death due to inhibition of photosynthesis [8]. Fruit yield and quality may be reduced in infected plants due to disease-induced sunscald, incomplete ripening, or poor storability [9].

Genetic resistance is an important tool for controlling powdery mildew on squash and pumpkin. Although regular foliar applications of fungicide can be used to manage the disease, fungicide-resistant strains of *P*. *xanthii* have reduced or eliminated the efficacy of many formerly effective fungicides [8, 10, 11]. Additionally, the most effective fungicides can be costly, especially when used repeatedly over the course of a long growing season [12]. Growers can deploy resistant varieties as part of an integrated management approach that requires less frequent, effective, and expensive fungicide applications [13]. Organic growers rely even more heavily on robust genetic resistance. Out of 105 respondents from a survey of vegetable farmers in the northeastern US who managed at least part of their farm in accordance with organic standards, 89% responded that genetic resistance to powdery mildew on cucurbits was important, and 37% said that genetic resistance to powdery mildew should be considered a critical priority of breeding programs [14].

To date, genetic resistance to powdery mildew has never been identified in *C*. *pepo*, and is found in only a few wild accessions of *C*. *moschata*. In a screen of the entire USDA collection of *C*. *pepo* during the late 1960s, none of the 292 accessions were resistant *[15]*. More recent evaluations of cultivars and accessions belonging to the USDA *C*. *pepo* collection grown under field-infected and growth chamber-inoculated conditions have resulted in the identification of accessions with partial resistance, although none with a degree of resistance that is alone sufficient for control [16–18]. Additionally, robust resistance to powdery mildew in *C*. *pepo* has not been reported from accessions held internationally. For *C*. *moschata*, accessions with resistance have been reported, but resistance from these sources is not common in mainstream commercial cultivars [4, 15, 19–22].

**Fig 1:**
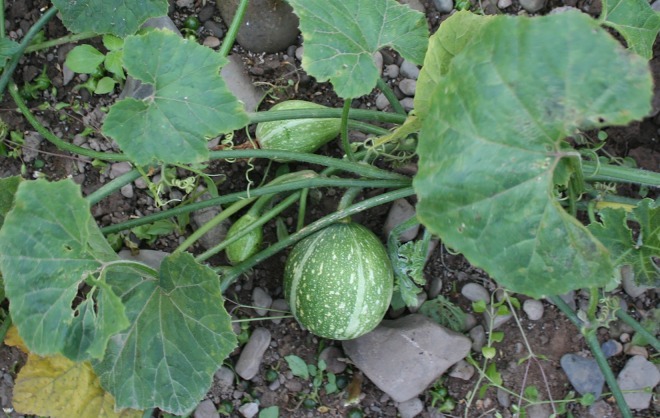
*Cucurbita okeechobeensis* subsp. *martinezii*. The wild inedible gourd, native to the Gulf Coast of Mexico [35], is depicted growing in Ithaca, NY. *C*. *okeechobeensis* subsp. *martinezii* is central in the *Cucurbita* clade and interfertile with other *Cucurbita [36]*. *C*. *okeechobeensis* subsp. *martinezii* is the original source of powdery mildew resistance now found in *C*. *pepo*.

Resistant wild *Cucurbita* species with which *C*. *pepo* and *C*. *moschata* are sparingly cross-compatible have been used to introgress resistance genes into cultivated material [23]. The wild *Cucurbita* species *C*. *lundelliana* contains a dominant resistance gene that was introgressed into *C*. *pepo* through a *C*. *moschata* bridge [24–27]. Cultivars with these introgressions have not been commercialized, however, due to linkage drag associated with the introgression and incompleteness of resistance in cultivated backgrounds [4, 20]. A breakthrough occurred when the resistance gene *Pm-0*, from the wild species *C*. *okeechobeensis* subsp. *martinezii* (Fig 1), was successfully introgressed into squash and pumpkin at Cornell University. This was achieved first in *C*. *moschata* with a cross to ‘Butternut’ beginning in 1974, and later in *C*. *pepo* through the interspecific hybrid cross: (((*C*. *pepo* ‘Yankee Hybrid’ x *C*. *moschata* ‘Butternut’) x 'Yankee Hybrid) x (*C*. *moschata* ‘Butternut 23’ x *C*. *okeechobeensis* subsp. *martinezii* F_1_)) [4, 20, 28–30]. Following the initial crosses, the gene was incorporated into the open-pollinated *C*. *moschata* butternut cultivars ‘Bugle’ and ‘PMT Large Butternut’ and into open-pollinated cultivars of multiple morphotypes of both cultivated *C*. *pepo* subspecies. These included: ‘Success PM’, ‘PMR Bush Delicata’ and ‘Sweet REBA’, representing the straightneck, delicata, and acorn morphotypes, respectively, in the subspecies *C*. *pepo* subsp. *texana*, and ‘Romulus’, ‘PMR Caserta’, ‘Improved Costata’, and ‘PMR Naked Seeded’, representing the zucchini, vegetable marrow, cocozelle, and pumpkin morphotypes, respectively, in the subspecies *C*. *pepo* subsp. *pepo* [31, 32]. These Cornell cultivars or their progenitors have been used widely by other public and private breeding programs. At present, the *Pm-0* gene is responsible for resistance in nearly all powdery mildew resistant (PMR) commercial cultivars of *C*. *moschata* and *C. pepo [20]*, barring the possible exception of certain cultivars from Hollar Seeds [33]. The inheritance of *Pm-0* in most cultivated backgrounds is incompletely dominant. In many contexts, even without conferring complete resistance, the *Pm-0* gene in the homozygous or even heterozygous condition in *C*. *pepo* has been adequate for practical disease control [4, 22, 28, 34].

Resistant inbred *C*. *pepo* cultivars which contain the *Pm-0* introgression but are otherwise genetically diverse as a result of directional breeding efforts can be considered a community-generated shared-trait introgression panel. When combined with susceptible and especially heirloom cultivars (for this study defined as those pre-dating the *Pm-0* introgression event), these cultivars represent a powerful resource for mapping *Pm-0*. With the shared-trait introgression panel mapping approach, molecular markers are identified that define interspecific differences, *e*.*g*. markers that are monomorphic between diverse heirloom *C*. *pepo* cultivars, but polymorphic between the heirloom group and *C*. *okeechobeensis* subsp. *martinezii*. Subsequently, the genotypes for these markers are determined for all cultivars. Genomic regions in modern cultivars that contain alleles identical to the wild species are presumed derived from the wild species. Any wild species-derived introgression common among resistant cultivars becomes a candidate interval for the gene of interest. In the case of single, historic, and widely used alleles such as *Pm-0*, the potential for historical recombination events in at least some cultivars to have reduced the size of the candidate interval around the gene of interest is high, barring chromosomal inversions or other rearrangements present in the region containing the introgression. Previously, this approach has been used to map other major resistance genes derived from wild species in tomato [37, 38]. Our study has advantages over previous efforts in that only one gene from one wild donor species is known to be widespread among current cultivars for the trait of interest. Additionally, the original source of resistance is still available, pedigree records tracing *Pm-0* back to its original donor exist for a suite of university-bred diverse cultivars, and high-throughput genotyping enables saturation of the genome with high-density molecular markers.

Genotyping-by-sequencing (GBS), which has been used to genotype other cucurbits [39], is an increasingly popular and cost-effective option for the *de novo* generation of thousands of high-density single nucleotide polymorphism (SNP) markers. In brief, GBS is the sequencing of multiplexed reduced-representation libraries that are generated by the enzymatic digestion of whole genomic DNA [40]. GBS is highly flexible to user requirements in order to achieve a read-depth sufficient for SNP-calling in populations of different types and genomes of varying sizes. Additionally, an array of restriction enzymes can be used to enrich for regions containing particular DNA patterns, including methylation-sensitive enzymes that enrich for non-repetitive, gene-rich genomic regions [41].

The objective of this research was to map the location of *Pm-0*, the primary resistance gene in *C*. *pepo*, through introgression mapping of a shared-trait introgression panel. Our results were validated by association mapping in a panel of *C*. *pepo* cultivars, and by testing the predictive ability of *Pm-0*-linked GBS SNP markers in an independent F_2_ population from a cross of *C*. *okeechobeensis* subsp. *martinezii* PI 532363 x *C*. *moschata* ‘Burpee’s Butterbush’. Finally, we developed CAPS markers predictive of powdery mildew resistance from *Pm-0* in both *C*. *pepo* and *C*. *moschata* backgrounds that can be used for marker-assisted breeding efforts in further development of powdery mildew-resistant squash and pumpkin cultivars.

## Materials and Methods

### Plant Material

#### Introgression Mapping

Accessions and cultivars from three *Cucurbita* species were used to map the *Pm-0*-containing introgression. The original source of *C*. *okeechobeensis* subsp. *martinezii*, now PI 406680 [42], was regenerated from Cornell seed stocks and used to define “wild” alleles for SNP markers. A set of six *C*. *pepo* heirloom cultivars advertised in seed catalogs prior to the introgression of *Pm-0* into *C*. *pepo* and belonging to multiple morphotypes and subspecies were used to define “*C*. *pepo*” alleles for SNP markers. The heirlooms and morphotypes were: ‘Black Beauty’ (zucchini), ‘Green Bush Vegetable Marrow’ (vegetable marrow), ‘Costata Romanesco’ (cocozelle), ‘Spirit’ (pumpkin), ‘Table King’ (acorn), and ‘Early Golden Summer Crookneck’ (crookneck). The shared-trait introgression panel consisted of a set of nine Cornell lines of *C*. *pepo* and *C*. *moschata* described in the introduction and listed in Table 1. Alleles in the resistant *C*. *moschata* cultivars were compared with the powdery mildew-susceptible *C*. *moschata* heirloom ‘Burpee’s Butterbush’.

**Table 1:** Germplasm used for introgression and association mapping of *Pm-0*.

Name	Sp.	Subsp.	Type	Source	PMR	Name	Sp.	Subsp.	Type	Source	PMR
PI 406680	o	mar	go	Cornell	R	PL5124-1	p	pepo	pn	Rupp	UD
**Bugle**	m		bn	Cornell	R	Segev F_1_	p	pepo	vm	High Mowing	R
**PMT Lg. Butternut**	m		bn	Cornell	R	Caliph F_1_	p	pepo	vm	Harris Moran	IR
**Success PM**	p	tex	sn	Cornell	R	Citlali F_1_	p	pepo	vm	Harris Moran	IR
**PMR Bush Delicata**	p	tex	de	Cornell	R	Hurakan F_1_	p	pepo	vm	Harris Moran	IR
**Sweet REBA**	p	tex	ac	Cornell	R	Cha-Ching F_1_	p	pepo	zu	High Mowing	R
**Romulus**	p	pepo	zu	Cornell	R	Emerald Delight F_1_	p	pepo	zu	Territorial	R
**PMR Caserta**	p	pepo	vm	Cornell	R	Dunja F_1_	p	pepo	zu	Johnny's	IR
**Improved Costata**	p	pepo	cz	Cornell	S	Elegance F_1_	p	pepo	zu	Harris Moran	IR
**PMR Nkd. Sd. Pkn.**	p	pepo	pn	Cornell	R	Golden Glory F_1_	p	pepo	zu	Johnny's	IR
***Black Beauty***	p	pepo	zu	Baker Creek	S	Midnight Lightning	p	pepo	zu	High Mowing	IR
***Green Bush Veg*. *Mw*.**	p	pepo	vm	Baker Creek	S	Paycheck F_1_	p	pepo	zu	Stokes	IR
***Costata Romanesco***	p	pepo	cz	High Mowing	S	Payroll F_1_	p	pepo	zu	Stokes	IR
***Spirit***	p	pepo	pn	Jung	S	Preference F_1_	p	pepo	zu	Harris Moran	IR
***Table King***	p	tex	ac	Olds	S	Prestige F_1_	p	pepo	zu	Harris Moran	IR
***Early Gn*. *Smr*. *Cknk*.**	p	tex	cn	Baker Creek	S	Quirinal F_1_	p	pepo	zu	Stokes	IR
***Burpee's Butterbush***	m		bn	Rupp	S	Sebring F_1_	p	pepo	zu	Fedco	IR
Camaro F_1_	p	pepo	pn	Hollar	R	Spineless Perfctn. F_1_	p	pepo	zu	Johnny's	IR
Charisma F_1_	p	pepo	pn	Johnny's	R	Wildcat F_1_	p	pepo	zu	Harris Moran	IR
Hijinks F_1_	p	pepo	pn	Osborne	R	Partenon F_1_	p	pepo	zu	Johnny’s	IR
Mustang F_1_	p	pepo	pn	Hollar	R	Ambassador F_1_	p	pepo	zu	Osborne	S
WeeeeeOne F_1_	p	pepo	pn	Rupp	R	Caserta	p	pepo	zu	Baker Creek	S
Bumpkin F_1_	p	pepo	pn	Harris	IR	Zucchini Elite F_1_	p	pepo	zu	Harris	S
Diablo F_1_	p	pepo	pn	Fedco	IR	Honey Bear F_1_	p	tex	ac	Johnny's	R
Gargoyle F_1_	p	pepo	pn	Harris	IR	Sugar Dumpling F_1_	p	tex	ac	High Mowing	R
Gladiator F_1_	p	pepo	pn	Harris Moran	IR	TipTop PMR F_1_	p	tex	ac	Johnny's	R
Gold Dust F_1_	p	pepo	pn	Rupp	IR	Autumn Delight F_1_	p	tex	ac	Osborne	IR
Iron Man F_1_	p	pepo	pn	Harris	IR	Royal Ace PM F_1_	p	tex	ac	Harris Moran	IR
Magic Lantern F_1_	p	pepo	pn	Harris	IR	Table Star F_1_	p	tex	ac	Rupp	IR
Magician F_1_	p	pepo	pn	Harris Moran	IR	Table Treat F_1_	p	tex	ac	Rupp	IR
Merlin F_1_	p	pepo	pn	Osborne	IR	Taybelle PM F_1_	p	tex	ac	Stokes	IR
Mischief F_1_	p	pepo	pn	Harris Moran	IR	Celebration F_1_	p	tex	ac	Rupp	IR
Owl's Eye F_1_	p	pepo	pn	High Mowing	IR	Ebony	p	tex	ac	Reimer	S
Prankster F_1_	p	pepo	pn	Rupp	IR	Sweet Lightning F_1_	p	tex	ac	Rupp	IR
Warlock F_1_	p	pepo	pn	Harris	IR	Delicata	p	tex	de	Baker Creek	S
Rival PMR F_1_	p	pepo	pn	Johnny's	IR	Delta F_1_	p	tex	cn	Territorial	IR
Chucky F_1_	p	pepo	pn	Johnny's	S	Sunglo F_1_	p	tex	cn	Osborne	IR
G.bumps Spr. Frk. F_1_	p	pepo	pn	Territorial	S	Gold Star F_1_	p	tex	cn	Osborne	IR
Howden	p	pepo	pn	High Mowing	S	Dk. Gn. Scall. F_1_	p	tex	sc	High Mowing	R
Sorceror F_1_	p	pepo	pn	Harris Moran	S	Yellow Scallopini F_1_	p	tex	sc	High Mowing	IR
PL3602-2	p	pepo	pn	Rupp	UD	Cheetah F_1_	p	tex	sn	Harris Moran	IR
PL3517-3	p	pepo	pn	Rupp	UD	Cougar F_1_	p	tex	sn	Harris	S
PL3885-1	p	pepo	pn	Rupp	UD						

*Cucurbita* spp. used for *Pm-0* mapping: *C*. *okeechobeensis* subsp. *martinezii* PI 406680, the original source of *Pm-0*, the Cornell-bred shared-trait introgression panel (bolded and underlined), *Cucurbita* heirlooms (bolded and italicized), and assorted *C*. *pepo* cultivars. For species (“Sp.”), p = *C*. *pepo*, m = *C*. *moschata*, o = *C*. *okeechobeensis* subsp. *martinezii*. For *C*. *pepo* subspecies (“Subsp.”), pepo = *C*. *pepo* subsp. *pepo*, tex = *C*. *pepo* subsp. *texana*. For morphotype (“Type”), ac = acorn, bn = butternut, cn = crookneck, cz = cocozelle, de = delicata, go = gourd, pn = pumpkin, sc = scallop, sn = straightneck, vm = vegetable marrow, zu = zucchini. Subspecies and morphotypes are as defined by Paris et al. and Gong et al. [31, 32]. For “PMR”, resistance phenotypes are listed as described/inferred from the vendor’s website. R = resistant, IR = intermediately resistant (sometimes described as “tolerant”), S = susceptible, UD = undefined. These classifications were used for cultivar selection only and not for downstream analysis. Selected cultivars are abbreviated as follows: PMT Lg. Butternut = Large Powdery Mildew Tolerant Butternut, PMR Nkd. Sd. Pkn. = Powdery Mildew-Resistant Naked-Seeded Pumpkin, Green Bush Veg. Mw. = Green Bush Vegetable Marrow, Early Gn. Smr. Cknk. = Early Golden Summer Crookneck, G.bumps Spr. Frk. F_1_ = Goosebumps Super Freak F_1_, Spineless Perfctn. F_1_ = Spineless Perfection F_1_, Dk. Gn. Scall. = Dark Green Scallopini.

#### Association Mapping

The *Pm-0-*containing genomic region identified through introgression mapping was confirmed by association mapping in a panel of 81 *C*. *pepo* cultivars that included 68 *C*. *pepo* commercial cultivars in addition to the seven Cornell-bred *C*. *pepo* cultivars in the shared-trait introgression panel and the six heirlooms used for introgression mapping. The species, subspecies, morphotype, seed source, and putative resistance based on catalog description of each cultivar are listed in Table 1.

#### Biparental Population

A biparental F_2_ population consisting of 177 individuals from a cross between *C*. *okeechobeensis* subsp. *martinezii* PI 532363 and the powdery mildew susceptible *C*. *moschata* ‘Burpee’s Butterbush’ was used to generate a genetic map to anchor SNP markers, and to test *Pm-0-*linked SNPs for predictiveness of resistance in a segregating population.

### Genotyping

#### DNA Extraction

DNA was extracted and diluted in preparation for GBS. Two to three meristematic leaves from single plants of each cultivar, or in the case of the F_2_ population, from each plant, were collected in the field. Samples were then lyophilized for at least 48 hours. DNA was extracted using the frozen/lyophilized plant tissue protocol starting on page 35 of the 2012 Qiagen DNeasy Plant Handbook (https://www.qiagen.com/us/resources/resourcedetail?id=95dec8a9-ec37-4457-8884-5dedd8ba9448&lang=en) but eluted with 30 μL of Buffer AE twice for a final volume of 60 μL. Samples were then quantified using the Invitrogen Quant-iT PicoGreen kits. One microliter from each sample was pipetted into 198 μL of 1x TE buffer and 0.5 μL of 200x PicoGreen. Samples were quantified in a black, flat-bottomed 96-well plate with a SpectraMax plate reader using an excitation wavelength of 480 nm and emission wavelength of 520 nm. Fluorescence units were converted to concentrations based on a standard curve calculated using eight different concentrations of Lambda DNA from 0 to 200 ng/μL. DNA was diluted to a final concentration of 10 ng/μL.

#### GBS Library Preparation

Genotyping-by-sequencing was used to genotype all samples. 96-plex libraries were prepared according to the protocol described by Elshire et al. [40]. All distinct genotypes were sequenced individually except the parents of the F_2_ mapping population and *C*. *okeechobeensis* subsp. *martinezii* PI 406680, which were sequenced in replicate. The partially methylation-sensitive restriction enzyme ApeKI, which recognizes a degenerate five base pair sequence, was chosen for the digestion step due to its potential to enrich for gene-rich regions. Excess primer dimers in the library were removed using 1.8X volumes of the Agencourt AMPure beads (Beckman Coulter). Each GBS library was sequenced on one lane of a HiSeq 2000 Illumina Sequencing System.

#### Calling SNPs

SNPs were called using the TASSEL-GBS pipeline build 5.2.10 [43]. Bowtie2 was used to align Illumina reads to the *C*. *pepo* zucchini genome draft v3.2 pre-released by a joint effort of the Genomics and Bioinformatics and Cucurbits Breeding Groups of the COMAV–Polytechnic University of Valencia (www.cucurbigene.net). To accommodate formatting constraints within the TASSEL pipeline, the first 19 largest scaffolds in the draft genome were left unmodified, and all remaining scaffolds were concatenated into a superscaffold with 80 “N”s inserted between each of the original scaffolds. Default TASSEL pipeline parameters were used with the exception that the parameter “c” (minimum number of times a tag must be present to be output) was set at five for the MergeMultipleTagCount and TagCountToFastq plugins.

### Genetic Map Construction

A genetic map was created using stringently filtered markers called in the *C*. *okeechobeensis* subsp. *martinezii* PI 532363 x *C*. *moschata* ‘Burpee’s Butterbush’ F_2_ population in order to anchor markers for downstream analyses. Using a custom python script, genotypes represented by less than seven reads were converted to missing data in order to reduce errors associated with under-calling or the false identification of heterozygous loci, common problems for low-coverage loci [44, 45]. Seven reads is the minimum number required to call a heterozygote using at least two reads of the “less tagged allele” based on the binomial likelihood ratio employed in TASSEL and assuming a sequencing error rate of 1%, a conservative estimate for Illumina sequencing [43, 46]. TASSEL was subsequently used to filter SNPs by a minimum minor allele frequency of 0.25, a locus call rate of 0.95 and a taxa call rate of 0.85 [47]. SNPs characterized by different alleles between the parents were selected using the ABH Genotype plugin in TASSEL [48]. The package R/qtl in the R statistics environment was used to generate the genetic map [49, 50]. Duplicate individuals and markers were removed, as well as markers showing segregation distortion, as determined by a p-value less than 1 x 10^−8^. Recombination frequencies between all pairs of markers were estimated using the function “est.rf”. Linkage groups (LGs) were formed using the “formLinkageGroups” function with a maximum recombination frequency of 0.15 and minimum lod of 25. The single marker that was not placed on the 20 primary LGs was discarded. Markers were ordered on LGs with the “OrderMarkers” function, and marker order was evaluated over a sliding window of 6 using the “ripple” option. Linkage disequilibrium between all pairs of markers for each chromosome were plotted, and in regions visually suggestive of incorrect ordering, markers were manually reordered if the new order increased the LOD score and decreased the length of the LGs. Sixteen markers were removed that in the majority of individuals were flanked by non-like genotypes, and genotypes with a high probability of being errors as defined by an error LOD score greater than 2 using the “calc.errorlod” function were changed to missing data using a custom Perl script.

### Introgression Mapping

For each of the heirlooms and Cornell-bred shared-trait introgression panel cultivars, GBS marker genotypes were plotted along all 20 linkage groups using the genetic map to anchor markers with common SNP identification numbers. Alleles were shaded blue if the locus genotype was homozygous for the “wild” allele, identical to *C*. *okeechobeensis* subsp. *martinezii*, gray if the marker genotype was homozygous for the “*C*. *pepo”* allele, identical to all *C*. *pepo* heirlooms, or light blue if in the heterozygous state. Any markers that were not represented on the *C*. *okeechobeensis* subsp. *martinezii* PI 532363 x *C*. *moschata* ‘Burpee’s Butterbush’ F_2_ genetic map by common SNP ID numbers or that displayed interspecific monomorphism or intraspecific polymorphism were filtered out using the TASSEL ABH plugin and a custom Perl script. Missing genotypes that were doubly flanked by markers with identical genotype were imputed to the flanking genotype. Loci genotypes that were positioned at least 20 cM distant from an identical genotype, and which was positioned no more than 3 cM distant from flanking genotypes that were different to the locus under consideration but identical to each other, were considered errors and converted to the flanking genotypes.

After a genomic *Pm-0-*containing introgression region was identified, this region was mapped at higher resolution using all called SNP markers in the region ordered by their scaffold positions, regardless of whether the markers were represented in the genetic map. Markers were filtered by a locus call rate of 0.50 and missing genotypes were imputed using default settings in Beagle 4.0 [51]. SNPs defined by alternate alleles between *C*. *okeechobeensis* subsp. *martinezii* and *C*. *pepo* were selected as described for the whole genome introgression map. Marker genotypes were considered errors and converted to flanking genotypes if they were within 5 kb of flanking markers with different genotypes which were in turn part of a long string of identical marker genotypes that extended more than 10 kb in each direction. A *Pm-0*-containing candidate interval was identified by the common area of overlap between the introgressions in all resistant cultivars.

### *Pm-0* Validation by Association Mapping

**Fig 2:**
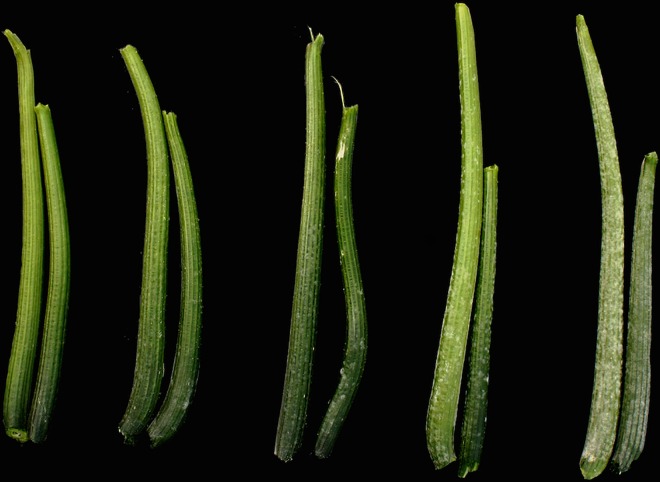
Petiole rating using a 1–5 scale. 1—No pathogen colonies visible on petioles. 2—A small number of colonies limited to the base of some petioles. 3—Colonies on nearly all petioles near the base, and extending halfway up the petiole. 4—Colonies on all petioles, extending the full length of the petiole to the leaf blade, but lacking colony density of fully susceptible cultivars, especially near the leaf blade. 5—All petioles covered with pathogen colonies from petiole base to the leaf blade at high density; most individual colonies have coalesced into larger colonies.

Association mapping was used to validate the *Pm-0-*containing genomic interval identified by introgression mapping. Cultivars were grown and phenotyped in Ithaca, NY in the summer of 2013. Cultivars were transplanted in six-plant plots in a randomized complete block design with three replicates. Plants were transplanted near a squash field with high loads of natural inoculum; disease pressure was increased two weeks after transplanting by inoculating a mixture of cultivars planted around the perimeter of the field and throughout the field at five row intervals with a suspension of *P*. *xanthii* conidia from nearby squash plants and diluted to 10,000 spores mL -1 in a .002% Tween 20 solution. The pathogen of powdery mildew was determined by amplifying and sequencing rRNA ITS4 and ITS5 regions as described by White et al. and aligning them to NCBI sequences in the non-redundant (nr) database (http://blast.ncbi.nlm.nih.gov/Blast.cgi) [52,53]. Early-fruiting summer squash cultivars were stripped of harvestable fruit on a weekly basis to remove resistance effects associated with maturity and fruit load. After six weeks, petioles of fully-expanded leaves were rated on a per-plot basis, averaged over three plots, using a scale described in Fig 2. Petiole ratings were chosen based on our previous observations in both cultivar panels and biparental populations that petiole symptoms at this stage of development were the most straightforward and reliable predictors of *Pm-0* dosage and presence/absence of powdery mildew resistance in the rest of the plant. In addition to “high”, “medium”, and “low” disease ratings, which might be expected for a single incompletely dominant gene, intermediary classifications were also included, which accounted for observed variations in the field and the likely presence of small-effect modifier genes.

For the analysis, we used a mixed linear model approach using the SUPER GWAS method as implemented in GAPIT, controlling for population structure with kinship and three principal components generated by the software [54, 55]. Markers from *C*. *pepo* cultivars were filtered for a minor allele frequency of 0.05 and a locus call rate of 0.50 and were drawn from scaffold locations within 30kb of markers identified on the F_2_ genetic map through common SNP ID numbers; they were subsequently assigned the genetic map position of their anchor marker using a custom python script. A Manhattan plot was generated in R using the qqman package [56].

### Refining the Interval

The *Pm-0*-containing genomic interval was reduced to a smaller interval by analyzing co-segregation between resistance phenotypes and selected marker genotypes for the shared-trait introgression panel and selected proprietary commercial cultivars. The interval was continuously narrowed based on absence of universal co-segregation of genotypes and phenotypes until an interval of 76.4 kb was reached with the flanking markers S9_1474683 and S9_1551065. CAPS primers were designed from 1000 bp sequences from the *C*. *pepo* draft 3.2 genome that surrounded GBS markers using Primer3Plus and filtered for single alignment to the genome using a custom python script [57]. The forward and reverse primers for S9_1474683 were: 5´-TGTCGCAGCATGACATCTAGTT-3´ and 5´-TGTCAGATATGGCGTCTGGATG-3´, respectively. The forward and reverse primers for S9_1551065 were 5´-ACGATCCATCCTCATTGACC-3´ and 5´-TGAGGACAGAGCAGCGAGTA-3´, respectively. CAPS markers were amplified with the following PCR reagents: 10 μL of 2 ng/μL DNA, 2 μL of 10x PCR buffer, 1 μL of 2.5 mM dNTPs, 0.25 μL of 10 μM forward primer, 0.25 μL of 10 μM reverse primer, 0.25 μL Taq polymerase, and 6.25 μL of sterile distilled water using the following thermocycler program: initial denaturation at 94°C for 3 minutes, 35 cycles of 94°C for 30 seconds, 55°C for 30 seconds, and 72°C for 90 seconds, and a final extension at 72°C for 15 minutes. PCR products were sequenced on an Applied Biosystems Automated 3730xl DNA Analyzer and analyzed with Sequencher version 4.9 to form consensus sequences [58]. The Sol Genomics Network (SGN) CAPS designer was used to select RsaI and PvuII as restriction enzymes to digest markers S9_1474683 and S9_1551065, respectively [59]. Samples were digested at 37°C for 2 hours using the following reagents: 10 μL of PCR product, 2 μL of 10x NEB CutSmart restriction buffer, 0.1 μL of 50 Unit/μL restriction enzyme, and 7.9 μL of sterile distilled water. The result was visualized on a 1.5% agarose gel.

### *Pm-0* Validation in a Segregating Population

GBS markers within the *Pm-0*-containing candidate genomic interval were validated within the *C*. *okeechobeensis* subsp. *martinezii* PI 532363 x *C*. *moschata* ‘Burpee’s Butterbush’ F_2_ population grown in Wauseon, OH by Rupp Seeds, Inc. Natural inoculum was prevalent in the field two months after transplanting, and ratings were taken approximately four months after transplanting near the end of the season. Petioles of F_2_ plants were scored with a binary rating, where 0 indicated no powdery mildew signs or symptoms, and 1 indicated presence of pathogen colonies and/or lesion symptoms. The *Pm-0-*containing interval identified by introgression mapping was divided into 10 bins spaced 50 kb apart. For the first GBS marker in each bin that showed no segregation distortion and a 95% call rate, a one-way ANOVA as implemented in the agricolae package in R was used to determine statistical difference between the genotype classes [60].

### Identification of Candidate Genes

The validated 76.4 kb *Pm-0-*containing genomic interval was aligned to the nr database by nucleotide BLAST using the NCBI web-interface and the megablast and discontiguous megablast options (http://blast.ncbi.nlm.nih.gov/Blast.cgi) [53].

### CAPS Marker Development and Validation for Marker-Assisted Selection

A CAPS marker in a putative NBS-LRR gene within the newly refined interval and displaying complete co-segregation of genotypes with phenotypes in the shared-trait introgression panel was developed for use in marker-assisted breeding using the same protocol used to develop the interval-defining CAPS markers. The forward and reverse PCR primers for the marker, labeled NBS_S9_1495924, were 5´-TCAACGGATATCTCCACCAAG-3´ and 5´-TACAGAGCAGCCTGGATGAGT-3´, respectively. The PCR products were digested with restriction enzyme HaeIII using the aforementioned described digest conditions. A secondary marker was developed as an additional resource. This marker was developed near the predicted *Cucumis melo* uncharacterized LOC103484742. The forward and reverse primers for this marker, S9_1539675 were 5´-ACTTAGAGAATGGTTCGACCTCTG-3´ and 5´-CTGGAGAGCTGTAAGTGAAGATCA-3´, respectively. The PCR products were digested with restriction enzyme MspI under the same restriction digest conditions as the previous enzymes.

## Results and Discussion

### Genotyping

GBS was used to call over 50,000 conservatively filtered markers in each species and in the F_2_ population, resulting in one of the largest SNP data sets to date for *Cucurbita*. Raw Illumina reads were trimmed to 64 bases and filtered for the presence of an expected cut site remnant, barcode sequence, and no missing bases with the TASSEL-GBS pipeline. For *C*. *pepo* cultivars, *C*. *moschata* cultivars, and the *C*. *okeechobeensis* subsp. *martinezii* PI 532363 x *C*. *moschata* ‘Burpee’s Butterbush’ F_2_ population, the number of filtered barcoded reads, reads aligning to the physical scaffolds, number of unique markers, average read depth, and missing data are reported in Table 2 for all GBS markers as well as for a subset with an average minimum read depth of five. GBS in 96-plex using the enzyme *Ape*KI is effective for generating high numbers of deep-coverage markers for the *Cucurbita* species included in this study.

**Table 2:** GBS sequencing read and marker statistics for genotyped *Cucurbita*.

		*C*. *pepo*	*C*. *moschata*	*C*. *okee*.	F_2_
**Individuals**		81	3(4)**	2(6)**	177
**Filtered Barcoded Sequencing Reads**		115,452,288	5,918,285	6,769,505	226,188,080
**Reads Aligned to Physical Scaffolds**		106,503,712	5,433,639	5,583,919	197,758,572
**All GBS Markers**		254,760	190,579	194,730	252,090
	Avg. Read Depth	5.62	7.87	5.59	4.91
	Proportion Missing Data	0.42	0.27	0.33	0.45
**GBS Markers with avg. min. read depth** ≥ **5**		61,090	63,058	53,796	57,151
	Avg. Read Depth	19.63	20.66	16.43	17.39
	Proportion Missing Data	0.04	0.02	0.03	0.07

*C*. *pepo* includes the cultivar panel. *C*. *moschata* includes ‘PMT Large Butternut’, ‘Bugle’, and ‘Burpee’s Butterbush’. *“C*. *okee*.*”* includes two *C*. *okeechobeensis* subsp. *martinezii* accessions: PI 406680, the original source of *Pm-0*, and PI 532363, one of the parents of the F_2_ population. The F_2_ population is derived from *C*. *okeechobeensis* subsp. *martinezii* PI 532363 and *C*. *moschata* ‘Burpee’s Butterbush’

** The number outside of the parentheses is the number of distinct genotypes. The number inside of the parenthesis includes the total number of individuals sequenced in the case where some genotypes were sequenced in multiple technical replicates. Values in the table represent all technical replicates.

### Genetic Map Construction

The *C*. *okeechobeensis* subsp. *martinezii* PI 532363 x *C*. *moschata* ‘Burpee’s Butterbush’ F_2_ population was used to generate a high-density genetic map for anchoring *C*. *pepo* SNP markers. The order of *C*. *pepo* markers based on a population derived from non-*C*. *pepo* parents was considered accurate based on previous reports describing synteny, no major chromosomal rearrangements, and high rates of marker transferability between *C*. *pepo* and *C*. *moschata* [61, 62], and the lack of any marker pairs in the map separated by large genetic distances which would indicate large chromosomal rearrangements between *C*. *moschata* and *C*. *okeechobeensis* subsp. *martinezii*. With stringent filtering conditions, our map yielded 2,669 markers over a total map distance of 2,199.2 cM, summarized in S1 Table, approximating the *C*. *pepo* map distance reported by Gong et al. for the only other *Cucurbita* map consisting of 20 LGs (1936 cM) [62], and the *C*. *pepo* map distance reported by Esteras et al. for the only other *Cucurbita* map generated with SNP markers (1740.8 cM) [63]. Identification numbers, LGs, and genetic map position for all markers are available in S2 Table. LGs are ordered by map distance. For marker ID numbers, the number following “S” corresponds to the scaffold of alignment from the *C*. *pepo* draft genome v3.2, with the exception of scaffold 20, which represents the “superscaffold” as described in the methods section. The number after the underscore corresponds to the base position of the relevant scaffold. For the 19 largest scaffolds of the *C*. *pepo* draft genome, only two scaffolds: 11 and 19, were not collinear on a single LG in our map. This could reflect chimeric scaffolds of the draft genome or rearrangement between *C*. *moschata* and *C*. *pepo*. In either case, the LGs containing these split scaffolds did not contain *C*. *okeechobeensis* subsp. *martinezii* introgressions, and were not important for downstream introgression or association mapping in this study.

### Introgression Mapping

**Fig 3:**
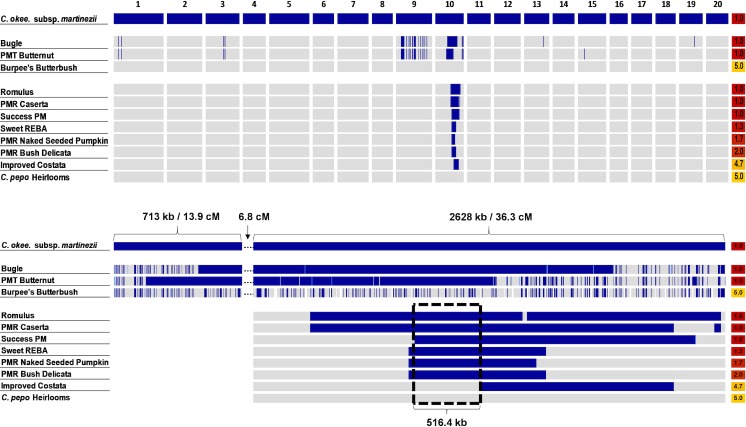
Introgression maps of Cornell-bred and heirloom *Cucurbita* inbreds. Genomic regions homozygous for the *C*. *pepo* alleles, as defined by the heirlooms, are shaded gray; genomic regions homozygous for the *C*. *okeechobeensis* subsp. *martinezii* alleles are shaded dark blue, and heterozygotes are shaded light blue. Cultivars are ordered based on petiole rating, from most resistant to least resistant, and secondly by the size of the largest and most prevalent *C*. *okeechobeensis* subsp. *martinezii* introgression on LG 10. (A) Whole Genome Map. LG 10 contains the *Pm-0*-containing introgression. (B) LG 10 Map. A dotted box appears around the 516.4 kb region of the introgression that all resistant cultivars share in common, indicating the putative interval for *Pm-0*. The region spans two scaffolds from the v.3.2 draft genome.

The *Pm-0*-containing introgression from *C*. *okeechobeensis* subsp. *martinezii* was mapped in a set of 17 Cornell-bred and heirloom *C*. *moschata* and *C*. *pepo* cultivars (Fig 3). Genotypes of 1,011 loci were plotted across 20 LGs; only loci present in the F_2_ genetic map and characterized by fixed, variant alleles between *C*. *okeechobeensis* subsp. *martinezii* and a set of six heirloom *C*. *pepo* cultivars were used. Heirloom cultivars, which were collectively used to define “*C*. *pepo*” allele genotypes, appeared true-to-type phenotypically and genotypically. One wild-derived introgression on LG 10 was common among all resistant cultivars and absent in all susceptible cultivars, identifying it as the *Pm-0-*containing region (Fig 3A). Of note is that the two Cornell-bred, powdery mildew-resistant *C*. *moschata* cultivars contain additional *C*. *okeechobeensis* subsp. *martinezii* introgressions absent in *C*. *moschata* ‘Burpee’s Butterbush’. Although these could contribute to resistance, it is likely that these introgressions are relicts from the breeding process, given that these cultivars are closely related to each other and are fewer generations removed from *C*. *okeechobeensis* subsp. *martinezii* than any of the *C*. *pepo* cultivars used in this study.

A higher resolution map of the introgression region illuminated a *Pm-0*-containing region (Fig 3B). The marker order of the physical scaffolds corresponding to this region on the genetic map agreed with the genetic map positions, and so all markers with a locus call rate greater than 0.5 were plotted and physical scaffold positions used, regardless of whether the marker was present in the genetic map. One side of the interval was defined by ‘Success PM’ using marker S9_1150923 and the other side of the interval was defined by marker S9_1667287 by ‘Improved Costata’, which displayed *C*. *okeechobeensis* subsp. *martinezii*-derived powdery mildew resistance in early generations of breeding but lost the resistance in later generations, as demonstrated by high petiole ratings. The cultivar retained some of the wild introgression, but not the portion containing *Pm-0*. The total size of the interval is 516.4 kb.

The small size of the candidate interval and the loss of resistance from ‘PMR Costata’ indicates that recombination events have occurred around the *Pm-0* gene as it has been incorporated into new cultivars. The capacity for recombination in this region to reduce the size of the wild introgression may be important to breeding efforts if the larger introgression contributes negatively to any non-disease-related horticultural and agronomic traits, as has been reported previously. For instance, *C*. *pepo* lines homozygous for the resistance gene have been reported as inherently lower-yielding when compared with susceptible commercial lines of the same fruit type [10]. Additionally, late-maturity has been associated with resistance in some cultivars [30]. However, these issues have been resolved in some cases by incorporating the resistance into new and especially highly productive backgrounds [20, 30], suggesting that either: large wild introgressions which contain alleles that retard yield or maturity can be decoupled from *Pm-0* through recombination, or that epistatic interactions between *Pm-0* or closely linked genes and certain genetic backgrounds may affect the pleiotropic expression of *Pm-0* for other non-disease resistance traits.

### *Pm-0* Validation by Association Mapping

**Fig 4:**
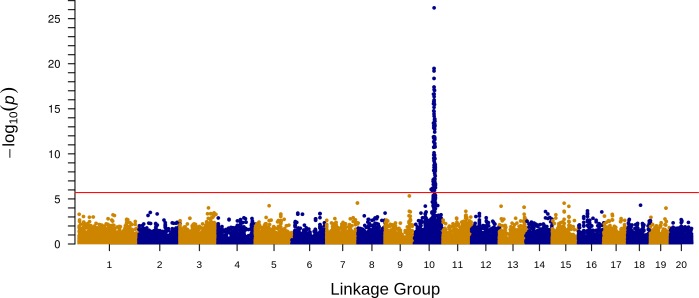
Mapping the *Pm-0* gene in the cultivar panel. Manhattan plot of negative log p-values for each marker across all 20 LGs. The threshold for significance was set at bonferonni-adjusted α = 0.05 of 1.96e-6. 145 markers on LG 10 are significant and the most highly significant markers fall within the *Pm-0* candidate interval identified through introgression mapping.

Association mapping validated the significance of the *Pm-0* candidate interval using a set of 25,446 markers. The squash cultivar panel was phenotyped amidst heavy and uniform disease pressure throughout the field in Ithaca, NY in 2013. The pathogen of powdery mildew was confirmed to be *P*. *xanthii* by 99% homology of sequenced rRNA ITS4 and ITS5 regions to NCBI sequences of *P*. *xanthii*. No phenotypic variation was observed among or between plots of any given cultivar that would indicate genetic segregation for powdery mildew resistance or any other trait. Average petiole ratings with standard error for Cornell-bred cultivars, heirloom cultivars, and commercial cultivars are listed in S3 Table. The SUPER method as implemented in GAPIT was used for mapping, and principal components and kinship were used to account for population structure, which clearly existed between the *C*. *pepo* subspecies. A clear peak on the Manhattan plot occurs in the *Pm-0* candidate interval (Fig 4), and the most significant p-value, 6.27e-27, is at marker S9_1551065 on LG 10.

The single GWAS signal, along with the lack of multiple wild-derived introgressions in the Cornell-bred resistance lines, suggests that powdery mildew-resistance in *C*. *pepo* varieties developed by U.S.-based seed companies is conferred largely by a single gene from *C*. *okeechobeensis* subsp. *Martinezii* PI 406680. However, resistance alleles from other sources may be important in some cultivars; petiole ratings between cultivars, even those representing the same market class and relative maturity, varied more than would be expected for a trait controlled entirely by a single incompletely dominant gene. Small-effect resistance-enhancing alleles may have been acquired in more resistant cultivars from susceptible ancestors that did not carry the alleles in the appropriate zygosity or genetic background for the expression of notable resistance [64].

### Refining the Interval

All 20 markers that were most significantly associated with powdery mildew resistance as determined by association mapping localized within the 516.4 kb *Pm-0*-containing interval identified through introgression mapping. To further refine the interval, CAPS markers were developed within the interval and genotyped in the shared-trait introgression panel and selected proprietary commercial cultivars until no recombinational breakpoints could be identified in any cultivar. The final interval of 76.4 kb was flanked by markers S9_1474683 and S9_1551065. This 76.4 kb interval was used for the identification of candidate genes.

### *Pm-0* Validation in Segregating F_2_ Population

SNP markers in the LG 10 *Pm-0-*containing interval from *C*. *pepo* were also associated with wild-derived resistance in an F_2_ population generated from a cross between *C*. *okeechobeensis* subsp. *martinezii* PI 532363, and *C*. *moschata* ‘Burpee’s Butterbush’. ANOVA tests of significance on four GBS markers from 50 kb bins within and immediately flanking the refined interval confirmed the effect of an incompletely dominant gene for powdery-mildew resistance in a 2^nd^ *C*. *okeechobeensis* subsp. *martinezii* accession–PI 532363 (Table 3).

**Table 3:** ANOVA of *Pm-0* in interspecific F_2_ population

Marker Name	Genotype	Genotype Average	Tukey's HSD
S9_1473058			
	A	0.6522	a
	H	0.5769	a
	B	0.4902	a
S9_1498203			
	A	0.7021	a
	H	0.561	ab
	B	0.4565	b
S9_1547588			
	A	0.7143	a
	H	0.5676	ab
	B	0.46	b
S9_1604471			
	A	0.7021	a
	H	0.5443	a
	B	0.5106	a

For every marker tested in and near the *Pm-0* candidate region, the class of individuals characterized by homozygous *C*. *moschata*-derived alleles (“A” genotypes) displayed higher scores for binary powdery mildew ratings on petioles when compared with the heterozygous class (“H” genotype) and the class with homozygous *C*. *okeechobeensis* subsp. *martinezii*-derived alleles (“B” genotypes). The markers inside of the refined interval were statistically significant at a *p-*value <0.05 as determined by a Tukey’s Honestly Significant Difference (HSD) test, while those outside of the refined interval were not statistically significant.

In phenotyping the interspecific F_2_ population, it was clear that in addition to *Pm-0* on LG 10, additional genes were contributing to resistance in the most disease-free individuals. Out of a total of 173 F_2_ individuals phenotyped, 75 were given a rating of 0, indicating that no *P*. *xanthii* colonies or powdery mildew symptoms were observed, even though disease pressure was high and ratings were taken at the end of a long season. The absence of single-gene Mendelian segregation patterns confirms observational data that the resistance in *C*. *okeechobeensis* subsp. *martinezii*, which is characterized as complete, is multigenic and complex. With replicated families and a quantitative rating system, it may be possible to identify some of these additional resistance alleles in the future, and the incorporation of new resistance alleles from *C*. *okeechobeensis* subsp. *martinezii* into *C*. *moschata* and *C*. *pepo* may be valuable to future squash breeding efforts. Although *Pm-0* continues to provide good control of powdery mildew in many trials of *C*. *pepo* in the US [34, 65, 66], additional control of powdery mildew may be needed in the future based on some recent reports indicating that the level of control provided by *Pm-0* appears reduced or eliminated relative to previous years [67, 68], potentially a result of the emergence of new races of *P. xanthii [69, 70]*.

### Identification of Candidate Genes

BLAST alignment of the 76.4 kb *Pm-*0-containing interval to the NCBI nr database yielded 14 putative genes, listed in S4 Table. Several putative genes in the interval are homologous to genes in other genera that are known to be involved in disease resistance. Of particular interest is the probable homolog of At5g66900, a NBS-LRR protein in *Arabidopsis thaliana* that contains a domain with similarity to the RPW8 locus that confers resistance to powdery mildew. In addition to *Arabidopsis*, NBS-LRR proteins have been found in powdery mildew resistance-associated regions in watermelon, a relative of *Cucurbita* spp. in the Cucurbitaceae family [71–74]. In addition to the putative NBS-LRR locus, numerous other candidates exist in the interval. At position 4, homology to a predicted peroxidase gene was identified from *C*. *melo*. Peroxidase gene clusters have been found to co-localize with basal powdery mildew resistance QTL in barley [75]. The predicted salicylic acid binding protein 2 (SABP2) was identified at position 44,701 from *C*. *melo*. Salicylic acid-induced defense responses, important for resistance to many biotrophic pathogens, have been described for *A*. *thaliana* against *G*. *cichoracearum*, one of the powdery mildew pathogens that also infects cucurbits [76, 77]. Finally, homology to a predicted Dof zinc finger was identified at position 52,057 from *C*. *melo*. Dof zinc finger proteins are known to have diverse functions, including response to infection [78]. A Dof zinc finger protein in *A*. *thaliana* has been shown to be associated with the regulation of defense genes as a response to signals from the salicylic acid pathway [79].

### Development of CAPS Markers for Marker-Assisted Selection

**Fig 5:**
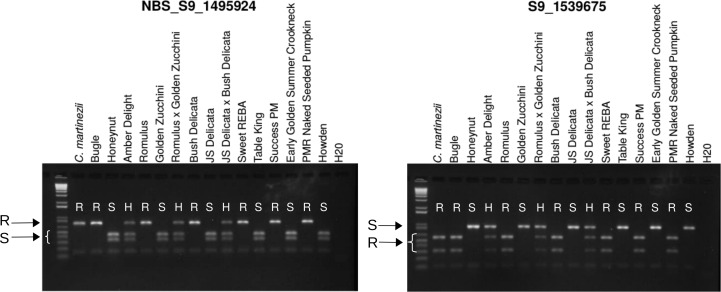
CAPS markers with complete co-segregation with *Pm-0* in a panel of susceptible and resistant cultivars. R = Homozygous for the *C*. *okeechobeensis* subsp. *martinezii-*derived resistance allele. S = Homozygous for the *C*. *pepo*/*C*. *moschata* susceptibility allele. H = Heterozygous. ‘Amber Delight’ is a hybrid of ‘Bugle’ and ‘Honeynut’. Left. NBS_S9_1495924 in a putative NBS-LRR gene with an *A*. *thaliana* powdery mildew resistance domain. Right. S9_1539675 in an unknown putative gene.

Two CAPS markers were developed for utility in marker-assisted selection. The first, NBS_S9_1495924, was located in the NBS-LRR gene. This marker distinguishes the resistance allele as a set of 134 and 759 bp fragments and the susceptible allele as a set of 134, 316, and 443 bp fragments. The marker fully co-segregates with the disease resistance phenotype as evaluated in the panel of Cornell-bred and heirloom *C*. *okeechobeensis* subsp. *martinezii* PI 406680, *C*. *moschata*, and *C*. *pepo* cultivars (Fig 5). A secondary marker with complete co-segregation, S9_1539675, is also reported (Fig 5). Both markers can be utilized for marker-assisted selection in breeding programs to screen and select for the presence of *Pm-0* in *C*. *pepo* and *C*. *moschata*.

## Conclusions

Using cultivars that comprised a shared-trait introgression panel and GBS to generate high-density genotype data, we have successfully mapped the major gene for powdery mildew resistance in squash, *Pm-0*, to a small genomic interval. The methods and tools presented here should be useful for elucidating other major genes, especially those derived from wild species, in squash and other crops. The CAPS markers presented here in addition to other sequence information should be useful to plant breeders seeking to employ marker-assisted selection towards the development of improved powdery mildew-resistant cultivars. Finally, we have identified a list of candidate genes that can be screened in future studies to definitively identify the *Pm-0* gene.

## Supporting Information

S1 Table Summary of *C*. *okeechobeensis* subsp. *martinezii* PI 532363 x *C*. *moschata* ‘Burpee’s Butterbush’ F_2_ linkage map. (XLSX)

S2 Table Marker ID numbers, linkage group membership, and positions for *C*. *okeechobeensis* subsp. *martinezii* PI 532363 x *C*. *moschata* ‘Burpee’s Butterbush’ F_2_ linkage map. (XLSX)

S3 Table Petiole ratings for *Cucurbita* germplasm used for introgression and association mapping. (XLSX)

S4 Table BLAST alignments reveal 14 putative genes within the 76.4 kb *Pm-0* candidate interval. (XLSX)
